# Dithienonaphthalene-Based Non-fullerene Acceptors With Different Bandgaps for Organic Solar Cells

**DOI:** 10.3389/fchem.2018.00427

**Published:** 2018-09-24

**Authors:** Meiqi Zhang, Yunlong Ma, Qingdong Zheng

**Affiliations:** ^1^State Key Laboratory of Structural Chemistry, Fujian Institute of Research on the Structure of Matter, Chinese Academy of Sciences, Fuzhou, China; ^2^University of Chinese Academy of Sciences, Beijing, China

**Keywords:** organic solar cell, non-fullerene, ladder-type structure, power conversion efficiency, bandgap

## Abstract

Compared to the traditional fullerene derivatives, non-fullerene acceptors show more tunable absorption bands as well as adjustable energy levels which are favorable for further PCE enhancement of organic solar cells. In order to enhance light-harvesting property of dithienonaphthalene (DTN)-based acceptors, we designed and synthesized two novel non-fullerene acceptors (DTNIF and DTNSF) based on a ladder-type DTN donor core flanked with two different acceptor units. In combination with a benchmark wide bandgap copolymer (PBDB-T), the best performance device based on DTNIF displayed a high PCE of 8.73% with a short-circuit current (*J*_sc_) of 13.26 mA cm^−2^ and a large fill factor (FF) of 72.77%. With a reduced bandgap of DTNSF, the corresponding best performance device showed an increased *J*_sc_ of 14.49 mA cm^−2^ although only a moderate PCE of 7.15% was achieved. These findings offer a molecular design strategy to control the bandgap of DTN-based non-fullerene acceptors with improved light-harvesting.

## Introduction

Organic solar cells (OSCs) have attracted increasing attention over the past decade due to their light-weight, mechanical flexibility, and potential low-cost (Facchetti, 2011; Liu et al., 2014; Rong et al., 2015). Bulk heterojunction (BHJ) OSCs featuring with an active layer of an electron acceptor material blended with an electron donor material, are widely used (Wu et al., 2011; Chen et al., 2013; Wang et al., 2014; Xu et al., 2015). In the early years' research on OSCs, fullerene derivatives, such as [6,6]-phenyl-C71-butyric acid methyl ester (PC_71_BM) and [6,6]-phenyl-C61-butyric acid methyl ester (PC_61_BM) have been used as the dominant electron acceptors due to their high electron mobilities and unique phase separation property when blended with rod-like donor materials (You et al., 2013; Ma et al., 2016). Although power conversion efficiencies (PCEs) of fullerene-based OSCs have surpassed 10% in single-junction OSCs (Liu et al., 2014; Chen et al., 2015; He et al., 2015; Zhang et al., 2015), the poor absorption in the visible region and the limited tunability in energy levels of the fullerene derivatives prevent a further PCE improvement of fullerene-based OSCs (Zhan et al., 2011). To break these limitations, emerging efforts have thus been devoted to designing non-fullerene acceptors which could have broader absorption, more adjustable energy levels and structural flexibility in comparison with the fullerene derivatives (Li et al., 2015; Lin et al., 2015; Nielsen et al., 2015; Liu et al., 2016, 2018; Qin et al., 2016; Zhang et al., 2016; Tang et al., 2017; Shen et al., 2018). Among the non-fullerene acceptors, small molecules with acceptor-donor-acceptor (A-D-A) configuration are popular in organic photovoltaic field because the HOMO and LUMO energy levels of A-D-A type molecules can be separately tuned by selecting suitable donor cores and acceptor terminals (Lin et al., 2015; Wu et al., 2015; Zhao et al., 2017). Using ladder-type angular-shaped dithienonaphthalene (DTN) as the donor unit and 2-(3-oxo-2,3-dihydro-1H-inden-1-ylidene)malononitrile (INCN) as the strong electron-withdrawing unit, we reported an A-D-A type non-fullerene acceptor (DTNIC8), recently, which exhibited a bandgap of 1.73 eV and a decent PCE of 9.03% (Ma et al., 2017b). In order to obtain DTN-based non-fullerene acceptors with an up-shifted LUMO energy level, we further used 5-(benzo[c][1,2,5]thiadiazol-4-ylmethylene)-3-ethyl-2-thioxothiazolidin-4-one as the weak electron-deficient group (Ma et al., 2017a). The resulting non-fullerene acceptor (DTNR) exhibited a similar bandgap of 1.72 eV but a much high-lying LUMO energy level of −3.75 eV which is beneficial for achieving a large *V*_oc_ for the corresponding PSC. Both the DTN-based acceptors showed relatively wide bandgaps with intense absorption bands in the range of 500–750 nm (Ma et al., 2017a,b). In order to improve the PCEs of OSCs based on wide bandgap donor materials, the bandgaps of non-fullerene acceptors based on DTN should be reduced further. For the A-D-A type non-fullerene acceptors, their bandgaps can be reduced by using stronger electron withdrawing groups as terminals (Zhao et al., 2017) and by extending π-conjugation length of the molecular backbone (Dai et al., 2017).

**Figure 1:**
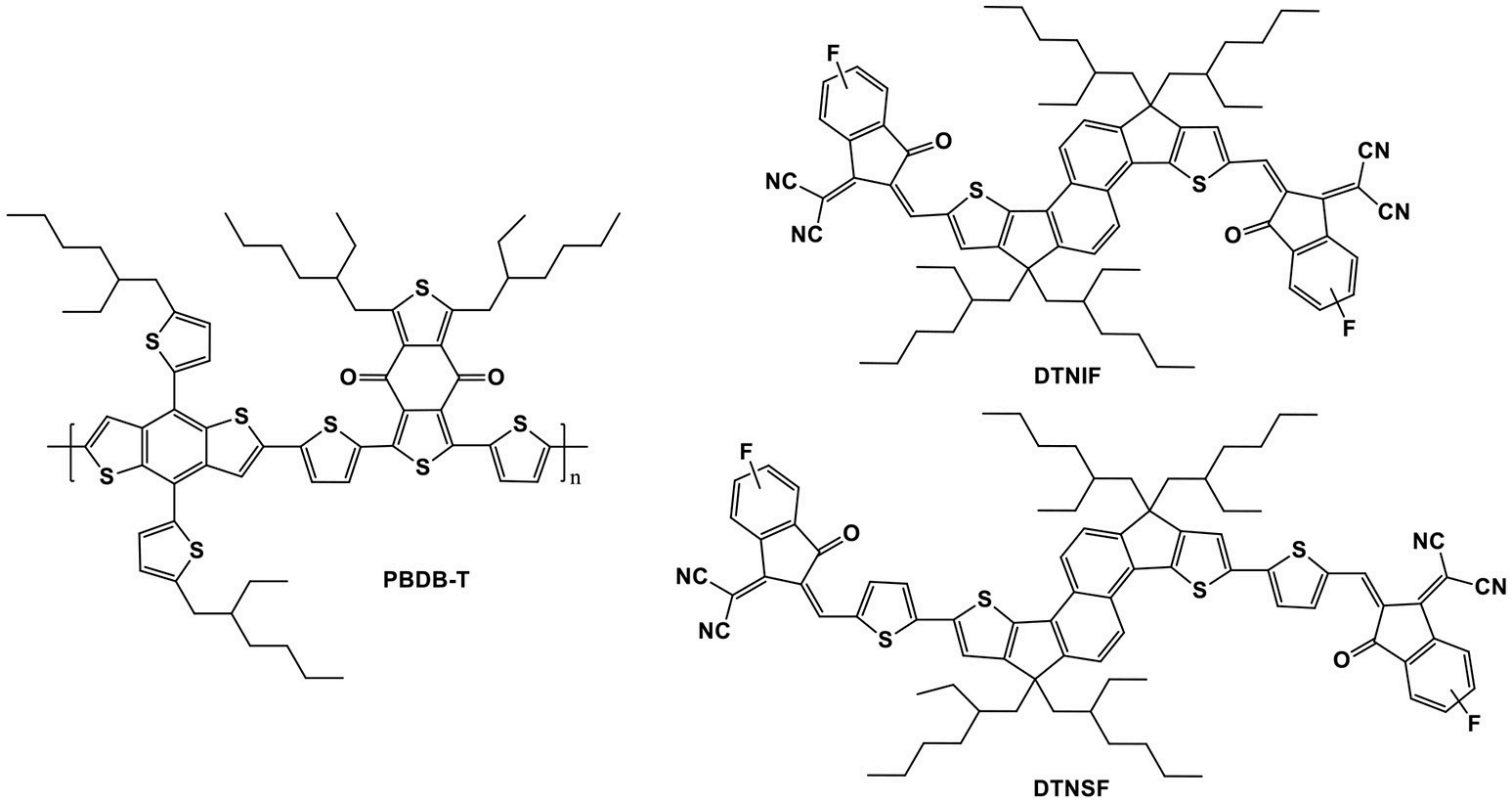
Chemical structures of PBDB-T and the target non-fullerene acceptors.

In this context, two novel DTN-based non-fullerene acceptors, DTNIF and DTNSF, were designed and synthesized by using a stronger electron withdrawing group of 2-(6-fluoro-3-oxo-2,3-dihydro-1H-inden-1-ylidene)malononitrile (INCNF), or by inserting two additional thiophene bridges in molecular backbone (shown in Figure 1). Inverted OSCs were fabricated by blending a typical wide bandgap copolymer (PBDB-T in Figure 1) with the DTN-based non-fullerene acceptors. The PBDB-T:DTNIF-based devices exhibited a PCE of 8.73% with a high FF of 72.77%, and a short circuit current (*J*_sc_) of 13.26 mA cm^−2^. However, the PBDB-T:DTNSF-based devices showed a moderate PCE of 7.15% with an increased *J*_sc_ of 14.49 mA cm^−2^ and a low FF of 54.62%. Moreover, we also studied effects of the terminal units on the bandgap, energy level, and charge transporting property of the DTN-based non-fullerene acceptors.

## Results and discussion

### Synthesis and characterization

**Scheme 1:**
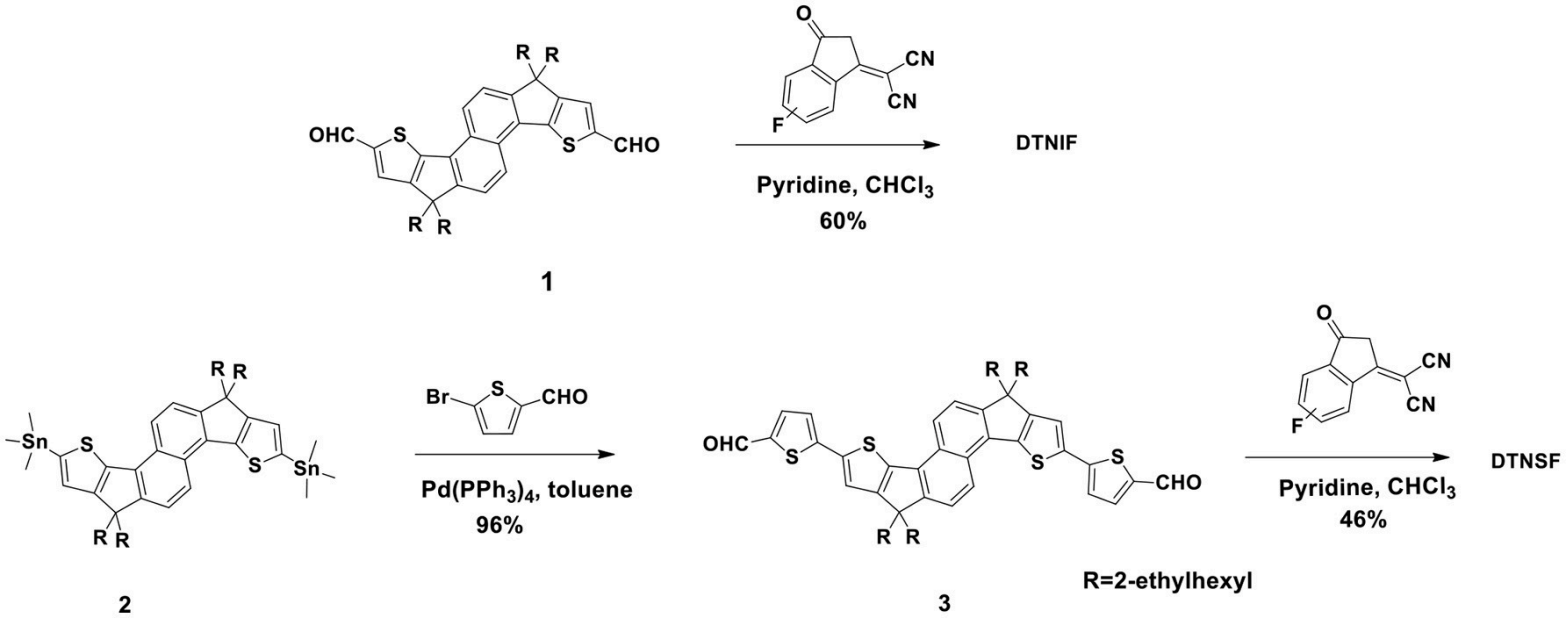
Synthetic routes of the non-fullerene acceptors.

The synthetic routes of DTNIF and DTNSF are shown in Scheme 1 and the synthetic details are described in the Experimental section. Compounds **1** and **2** were synthesized according to our earlier published methods (Ma et al., 2013, 2017b).

Compound **3** was obtained in 96% yield by the Stille coupling reaction between Compound **2** and 5-bromothiophene-2-carbaldehyde using Pd(PPh_3_)_4_ as the catalyst. A Knoevenagel condensation reaction between Compound **3** and INCNF afforded DTNSF in 46% yield. DTNIF was synthesized in 60% yield by using the same condensation reaction between Compound **1** and INCNF. The chemical structures of DTNIF and DTNSF were determined by using ^1^H NMR and high-resolution mass spectrometry. The purity of the acceptors was verified further by elemental analysis. All non-fullerene materials are soluble at room temperature in the traditional organic solvents, such as CH_2_Cl_2_, chlorobenzene, and CHCl_3_ etc.

### Optical and electrochemical properties

**Figure 2:**
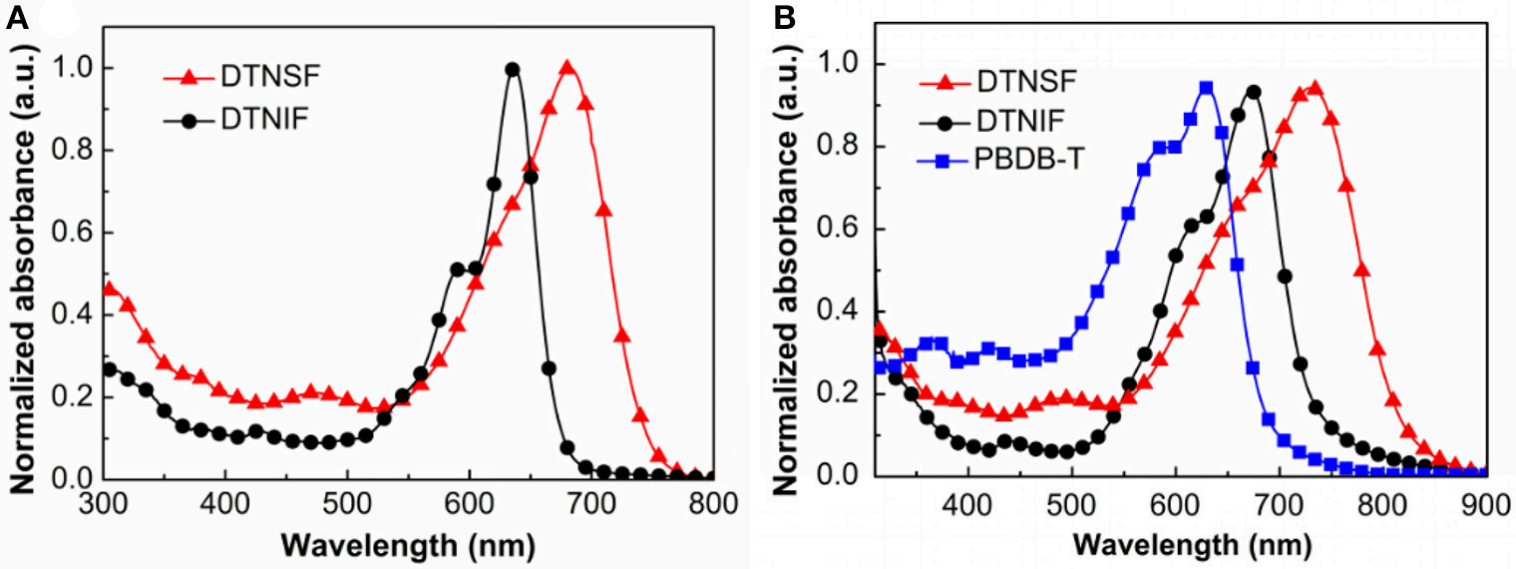
**(A)** Normalized UV–Vis absorption spectra of DTNIF and DTNSF in CHCl_3_. **(B)** Normalized UV–Vis absorption spectra of DTNIF, DTNSF, and PBDB-T in thin film.

The absorption properties of DTNIF and DTNSF were investigated in CHCl_3_ solution as well as in thin film. The obtained linear absorption spectra are shown in Figure 2 and the specific optical data are shown in Table 1.

**Table 1:** Optical and electrochemical properties of DTNIF and DTNSF.

**Molecules**	**ε [10^5^ M^−1^ cm^−1^]**	**$\lambda_{\text{max}}^{\text{solution}}$ [nm]**	**$\lambda_{\text{max}}^{\text{film}}$ [nm]**	**E${}_{\text{g}}^{\text{opt}}$ [eV]^a^**	**HOMO [eV]^b^**	**LUMO [eV]^c^**	**References**
DTNIF	1.8	636	672	1.63	−5.82	−3.92	This work
DTNSF	1.4	682	731	1.47	−5.52	−4.00	This work
DTNIC8	2.1	634	660	1.73	−5.91	−3.93	Ma et al., 2017b

a Estimated from the onset of the absorption spectra of thin films;

b E_HOMO_ = –(φ_ox_ + 4.82) eV;

c *E_LUMO_ = –(φ_red_ + 4.82) eV*.

In chloroform solution, DTNIF displayed a strong absorption band in the wavelength region of 500–700 nm with a clear shoulder peak at 600 nm which can be attributed to the intramolecular charge transfer from the electron donating core to the electron withdrawing terminals. Compared to DTNIF, DTNSF showed a red-shifted absorption band in the wavelength region of 520–780 nm which can be ascribed to its extended conjugation with two additional thiophene bridges. The maximum extinction coefficient of DTNIF (1.8 × 10^5^ M^−1^ cm^−1^ at 637 nm) was higher than that of DTNSF (1.4 × 10^5^ M^−1^ cm^−1^ at 682 nm). From solution to thin film, both the non-fullerene acceptors showed broader and red-shifted absorptions. The optical bandgaps estimated from their absorption edges were 1.63 eV and 1.47 eV for DTNIF and DTNSF, respectively, both of which are lower than the bandgap of DTNIC8 (1.73 eV in Table 1). With the standard A-D-A configuration in the molecular backbone, DTNIF exhibits a deep HOMO energy level of −5.82 eV. However, with the insertion of two additional thiophene units in the A-D-A backbone, the HOMO energy level of DTNSF increases to −5.52 eV together with a significant reduced bandgap of 1.47 eV which is mainly attributed to its extended π-conjugation in comparison with DTNIF. As shown in Figure 2B, DTNSF in thin film exhibits a more complementary absorption spectrum with PBDB-T in comparison that with DTNIF, suggesting a possible enhanced *J*_sc_ value for the DTNSF-based OSC.

**Figure 3:**
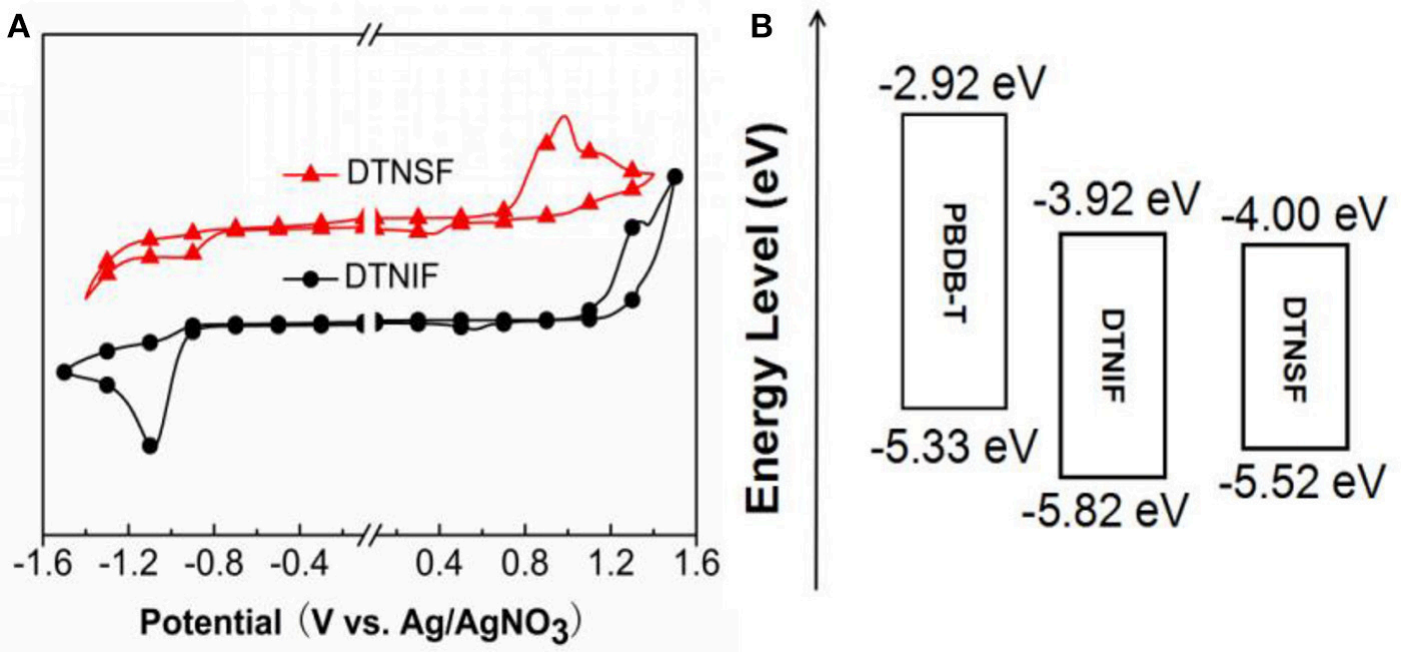
**(A)** Cyclic voltammograms of DTNIF and DTNSF. **(B)** Estimated energy level diagram of DTNIF, DTNSF, and PBDB-T.

The electrochemical properties of the non-fullerene acceptors were tested by electrochemical cyclic voltammetry (CV). Here, ferrocene was used as an internal reference, which has a HOMO level of −4.80 eV. The cyclic voltammograms are shown in Figure 3A and the corresponding data are listed in Table 1. According to their onset potentials, the HOMO/LUMO energy levels of DTNIF and DTNSF were calculated to be −5.82/−3.92 and −5.52/−4.00 eV, respectively. PBDB-T has HOMO and LUMO energy levels of −5.33 and −2.92 eV, respectively, which energetically matched with those of the acceptors (Figure 3B).

### Photoluminescence

**Figure 4:**
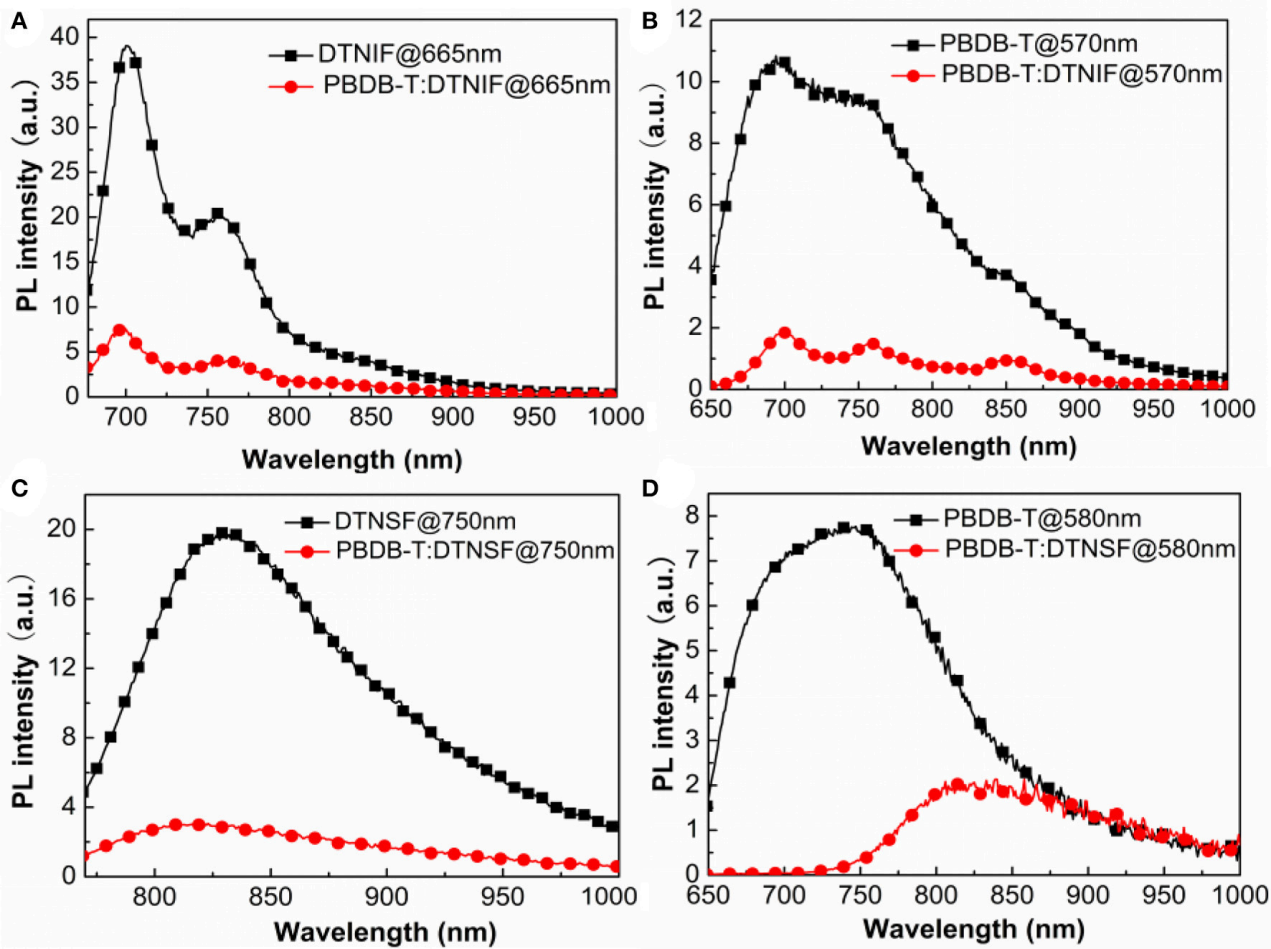
Fluorescence quenching experiments of PBDB-T:DTNIF **(A,B)** and PBDB-T:DTNSF **(C,D)** excited mainly by the acceptor **(A,C)** and the donor **(B,D)**.

In order to know the exciton dissociation as well as the charge transfer behaviors of donor/acceptor blends, photoluminescence (PL) quenching experiments were carried out and the results were shown in Figure 4. We selected 665 nm and 750 nm as the excitation wavelengths to respectively excite DTNIF and DTNSF in either pure or blend films. The donor/acceptor blend ratios were fixed at 1:1 by mol. As shown in Figures 4A,C, the strong emission of DTNIF at 700 nm and 760 nm (DINSF at 825 nm) in the blend film apparently quenched when compared to that in the pure film, demonstrating the efficient hole transfer from both the acceptors to PBDB-T (donor). As for the PL emission of PBDB-T (shown in Figures 4B,D), the PL intensities of PBDB-T:DTNIF and PBDB-T:DTNSF blend films decreased significantly in comparison with those of the pure PBDB-T film when excited at 570 and 580 nm, respectively. It suggested that there is efficient electron transfer from the PBDB-T donor to both the acceptors. These results demonstrated that both the non-fullerene acceptors and the polymer donor contribute to the photocurrent generation of the OSCs.

### Photovoltaic performance

**Figure 5:**
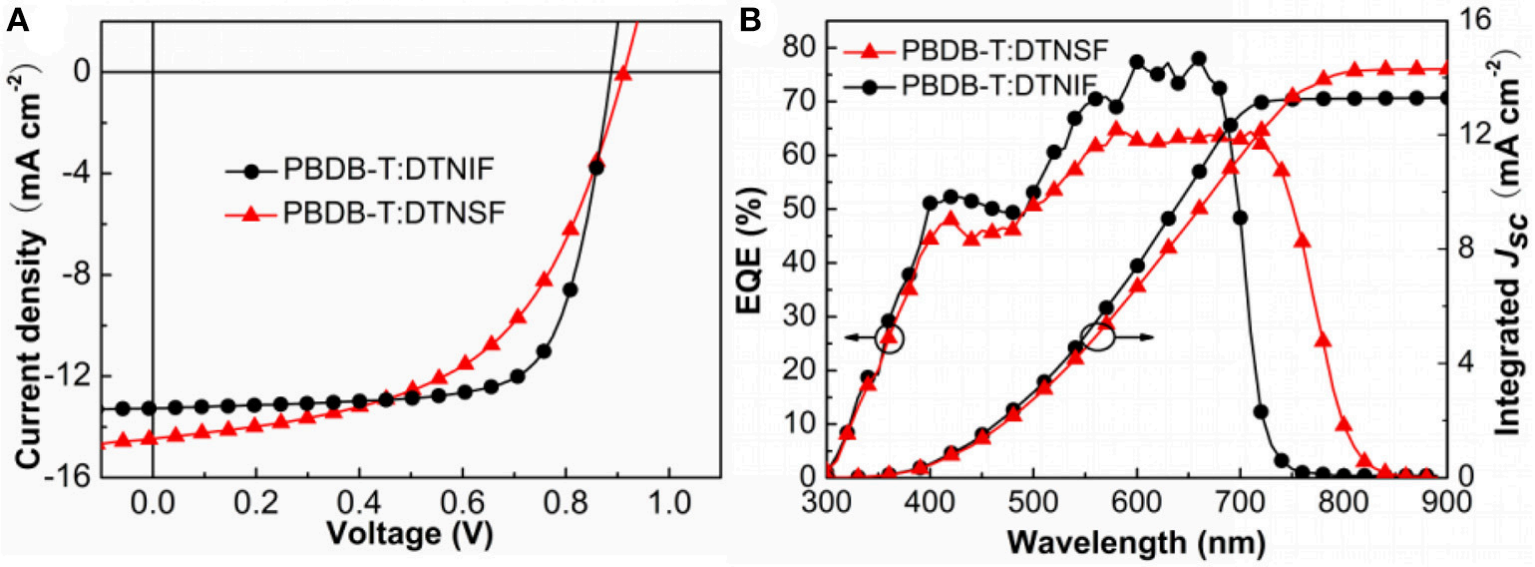
**(A)** *J*–*V* characteristics of the non-fullerene OSCs. **(B)** EQE and the corresponding integrated *J*_sc_ curves for the OSCs.

PBDB-T is a wide-bandgap polymer donor which has a strong absorption band in the wavelength range from 500 to 700 nm. The absorption bands of our non-fullerene acceptors generally match the absorption band of PBDB-T. Thus, we chose PBDB-T as donor to fabricate OSCs with an inverted device structure: indium tin oxide (ITO)/ZnO/donor:acceptor/MoO_3_/Ag. The active layers were spin-coated by using PBDB-T:acceptor (w/w, 1:1) blend solution in chlorobenzene (18 mg/mL) without any additives and post-treatments. The *J*–*V* curves of the best performance devices are shown in Figure 5A and detailed device parameters are summarized in Table 2.

**Table 2:** Photovoltaic properties of OSCs (under AM 1.5 G, 100 mW cm^−2^).

**Active layer**	***V*_oc_ (V)**	***J*_sc_ (mA cm^−2^)**	**FF (%)**	**PCE (%)**
PBDB-T:DTNIF	0.90	13.26	72.77	8.73 (8.65 ± 0.23)
PBDB-T:DTNSF	0.92	14.49	54.62	7.15 (6.94 ± 0.20)

Under simulated AM 1.5 G, 100 mW cm^−2^ illumination and the optimal device fabrication condition, the best performance OSC based on PBDB-T:DTNIF showed a PCE of 8.73% with a *V*_oc_ of 0.90 V, a *J*_sc_ of 13.26 mA cm^−2^ and a FF of 72.77%. Nevertheless, the best performance DTNSF-based device exhibited a PCE of 7.15% with a *V*_oc_ of 0.92 V and a lower FF of 54.62%. The lower FF was mainly resulted from the lower and less balanced hole and electron mobilities for the PBDB-T:DTNSF active layer. However, the *J*_sc_ of 14.49 mA cm^−2^ for the DTNSF-based device is larger than the PBDB-T:DTNIF-based counterpart owing to the red-shifted absorption of DTNSF. We noticed that the DTNSF-based device showed a slightly higher *V*_oc_ than the DTNIF-based device despite the fact that DTNIF possesses a higher LUMO level than DTNSF. Besides the energy gap (between HOMO of the donor and LUMO of the acceptor) which can affect the *V*_oc_ of corresponding device, other factors, such as recombination rate, reverse saturation current, carrier density, defect states and crystallinity, and charge-transfer states could also play an important role in influencing the *V*_oc_ (Elumalai and Uddin, 2016). Therefore, it is reasonable that the DTNIF-based device exhibited a relatively lower *V*_oc_ of 0.90 V.

As shown in Figure 5B, external quantum efficiency (EQE) spectra of the best performance devices were measured to ensure the accuracy of the PCE measurements. The device based on DTNIF has higher EQE values in the wavelength range of 300–750 nm with a maximum value of 78% at 660 nm. In contrast, the EQE spectrum edge of the best performance DTNSF-based device extended to 850 nm, which agrees with the absorption spectrum of the DTNSF blend film. The *J*_sc_ values obtained by integrating the EQE data with the solar spectrum (AM 1.5 G) were 13.26 and 14.21 mA cm^−2^ for DTNIF and DTNSF, respectively. The integrated values are in consistent with those from the *J-V* measurement within 2% error.

### Film morphology analysis

**Figure 6:**
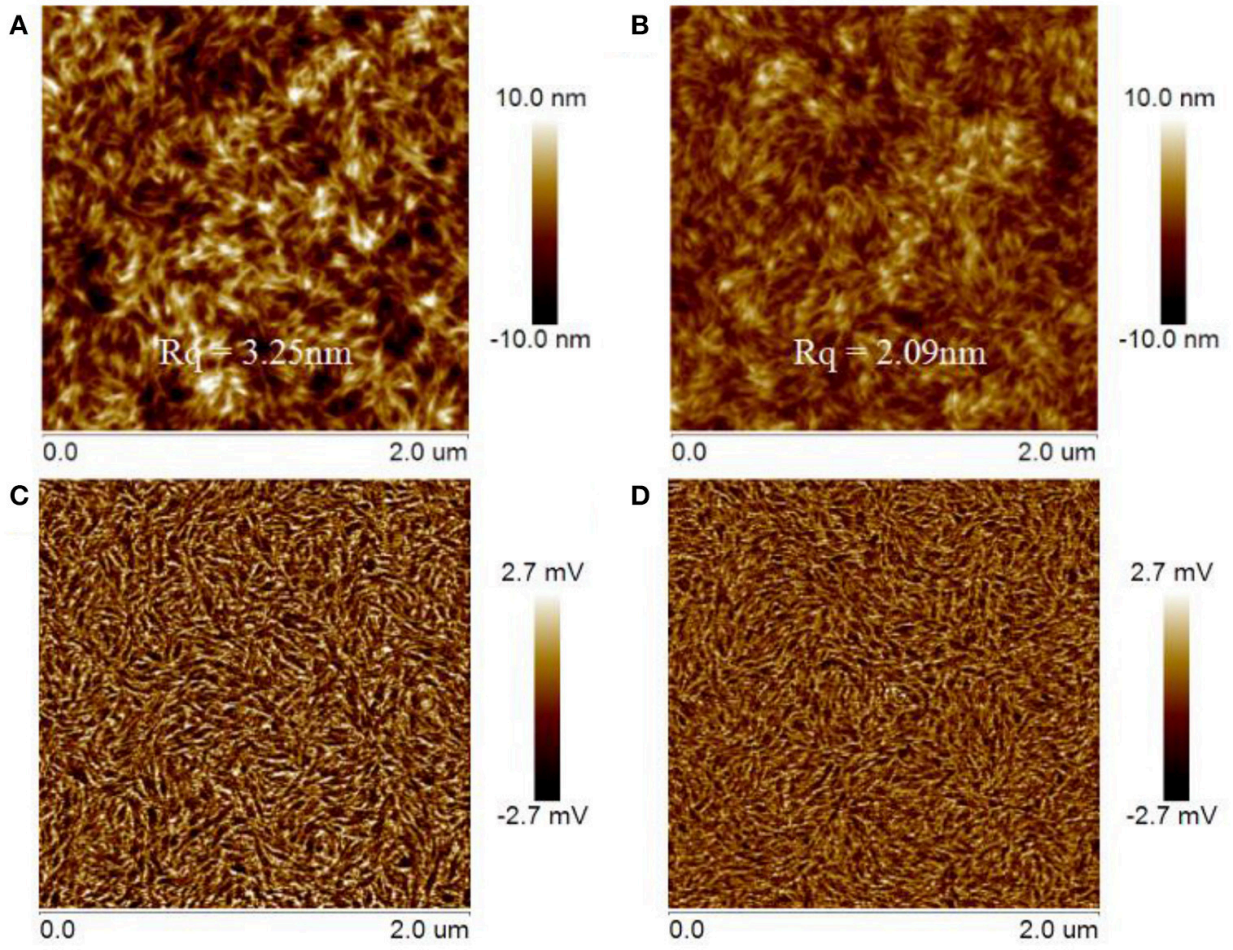
Tapping-mode AFM topography images **(A,B)** and phase **(C,D)** images of the PBDB-T:DTNIF **(A,C)** and PBDB-T:DTNSF **(B,D)** films.

Tapping-mode atomic force microscopy (AFM) was used to characterize the morphology of active layer that has an important influence on the performance of OSCs. The film samples for AFM analysis were prepared in identical fashion to those prepared for device fabrication in which the donor/acceptor blend ratios were fixed at 1:1 by mol. The obtained AFM images were presented in Figure 6. The AFM height images of the DTNIF and DTNSF-based blend films showed similar and apparently fibrillar structures (Figures 6A,B). However, the DTNSF-based blend shows smoother root-mean-square (RMS) roughness (R_q_) than the DTNIF-based blend. Compared to PBDB-T:DTNIF film with a R_q_ of 3.25 nm, the RMS roughness of PBDB-T:DTNSF film decreased to 2.09 nm which could be attributed to the smaller intramolecular twisted angel and greater coplanarity of DTNSF. As shown in the phase images (Figures 6C,D), fibrillar structure can also be observed in both the blend films. In comparison with PBDB-T:DTNSF blend film, PBDB-T:DTNIF film revealed fibrillar structures with larger sizes which will be favorable for efficient charge transport in the DTNIF-based devices as confirmed by their higher hole and electron mobilities.

**Figure 7:**
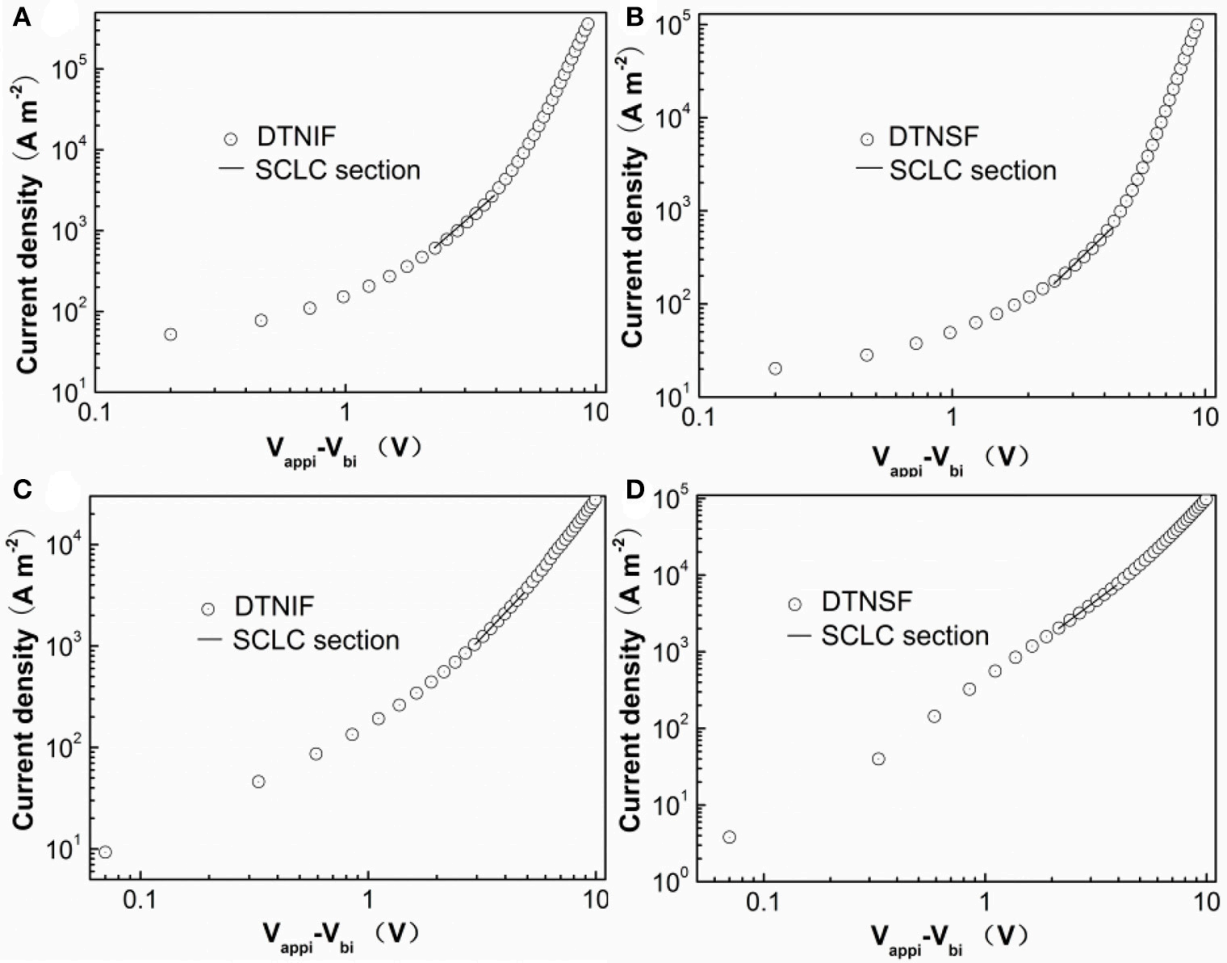
*J*–*V* characteristic for **(A,C)** DTNIF and **(B,D)** DTNSF-based **(A,B)** electron-only and **(C,D)** hole-only devices.

As mentioned above, the optimal morphology can enhance charge transport efficiency that will further affect the *J*_sc_ and FF of OSCs. We measured the electron (μ_*e*_) and hole (μ_*h*_) mobilities using the space charge limited current (SCLC) method with the device structures of ITO/ZnO/PBDB-T:acceptor/Ca/Al and ITO/PEDOT:PSS/PBDB-T:acceptor/Au, respectively. For both the hole- and electron-only devices, the donor/acceptor ratios for are fixed at 1:1 by mol. The *J-V* characteristics of the hole-only and electron-only devices are shown in Figure 7 and the mobility data are shown in Table 3. The μ_*e*_ and μ_*h*_ for the PBDB-T:DTNIF blend film were calculated to be 1.79 × 10^−5^ and 1.87 × 10^−5^ cm^2^ V^−1^ s^−1^, respectively, which far exceeded those for the PBDB-T:DTNSF film (μ_*e*_ = 6.70 × 10^−6^ and μ_*h*_ = 1.35 × 10^−5^ cm^2^ V^−1^ s^−1^). More balanced μ_*h*_/μ_*e*_ ratio of 1.04 was observed for the PBDB-T:DTNIF blend film when compared to a larger μ_*h*_/μ_*e*_ ratio of 2.01 for the PBDB-T:DTNSF blend. Thus, the higher and more balanced hole and electron mobilities of the PBDB-T:DTNIF blend can explain the higher FF of the resulting solar cell.

**Table 3:** Hole and electron mobilities of the SCLC devices based on two different active layers.

**Active layer**	***μ_*e*_* [cm^2^ V^−1^ s^−1^]**	***μ_*h*_* [cm^2^ V^−1^ s^−1^]**	***μ_*h*_*/*μ_*e*_***
PBDB-T:DTNIF	1.79 × 10^−5^	1.87 × 10^−5^	1.04
PBDB-T:DTNSF	6.70 × 10^−6^	1.35 × 10^−5^	2.01

## Conclusions

In summary, we have developed two novel non-fullerene acceptors, DTNIF and DTNSF, with different bandgaps. The introduction of F atom into the terminal group leads to a slightly narrow bandgap and a red-shifted absorption in the 500–750 nm region. To achieve a more complementary spectrum of non-fullerene acceptor with wide bandgap donor materials, such as PBDB-T, we further introduced two thiophenes as bridge units which lead to a more planar molecular configuration with an extended conjugation. Without any additive and post-treatment, the best performance DTNIF-based device exhibited a PCE of 8.73% with *V*_oc_ of 0.90 V, FF of 72.77% and *J*_sc_ of 13.26 mA cm^−2^. The best performance device based on DTNSF afforded an enhanced *J*_sc_ of 14.49 mA cm^−2^ although only a moderate PCE of 7.15% was obtained due to the decreased and unbalanced hole and electron mobilities of the DTNSF-based active layer. It should be noted that the device performance of DTNIF-based OSCs might be improved by selecting other donor polymers of more complementary absorption spectra.

## Experimental section

### Materials and characterization

All the solvents were purified and dried according to standard procedures. The donor polymer PBDB-T (99.9%) was bought from Solarmer Materials, Inc. Compounds **1** and **2** were prepared by using the reported procedure (Ma et al., 2013, 2017b).

Synthesis of **3**: Compound **2** (0.6 g, 0.56 mmol), 5-bromothiophene-2-carbaldehyde (0.32 g, 1.6 mmol) and Pd(PPh_3_)_4_ (30 mg, 0.03 mmol) were dissolved in 30 mL of degassed toluene in a two neck flask. After refluxing for 24 h under nitrogen, the mixture was cooled down to room temperature. Then the solvent was removed by evaporation, and the remaining residue was purified by column chromatography (silica gel) using petroleum ether/CH_2_Cl_2_ (3:1) as eluent. Finally, a dark brown solid (0.47 g, 96%) was obtained. ^1^H NMR (CDCl_3_, 400 MHz, ppm): 9.91 (s, 2H), 8.04 (d, *J* = 8.0 Hz, 2H), 7.75 (d, *J* = 8.0 Hz, 2H), 7.67 (d, *J* = 8.0 Hz, 2H), 7.43 (s, 2H), 7.37 (s, 2H), 2.17–2.04 (m, 8H), 1.01–0.52 (m, 60H). HRMS (MALDI) m/z: calc. for C_62_H_80_O_2_S_4_: 984.5017; found: 984.5027. Elemental analysis (%) calc. for C_62_H_80_O_2_S_4_: C, 75.56; H, 8.18; found: C, 75.79; H, 8.09.

Synthesis of **DTNSF**: To a solution of Compound **3** (200 mg, 0.2 mmol) in dry CHCl_3_ (30 mL), 2-(6-fluoro-3-oxo-2,3-dihydro-1H-inden-1-ylidene)malononitrile (340 mg, 1.6 mmol) were added. After degassing with nitrogen for 30 min, 0.15 mL of pyridine was added. The mixture was stirred at reflux for 24 h under nitrogen atmosphere. After the mixture was cooled to room temperature, it was poured into 100 mL of methanol. A precipitate was formed and filtered off which was further purified by using column chromatography (silica gel) with petroleum ether/CH_2_Cl_2_ (1:1) as the eluent. A dark green solid (130 mg, 46%) was obtained. ^1^H NMR (CDCl_3_, 400 MHz, ppm): 8.91 (d, *J* = 8.0 Hz, 2H), 8.42 (d, *J* = 8.0 Hz, 2H), 8.10 (d, *J* = 8.0 Hz, 2H), 8.01–7.97 (m, 2H), 7.86 (d, *J* = 8.0 Hz, 2H), 7.73–7.70 (m, 2H), 7.68 (d, *J* = 8.0 Hz, 2H), 7.50–7.45 (m, 4H), 2.20–2.09 (m, 8H), 1.05–0.54 (m, 60H). HRMS (MALDI) m/z: calc. for C_86_H_86_F_2_N_4_O_2_S_4_: 1,373.5689; found: 1,373.5674. Elemental analysis (%) calc. for C_86_H_86_F_2_N_4_O_2_S_4_: C, 75.18; H, 6.31; N, 4.08; found: C, 75.47; H, 6.20; N, 3.77.

Synthesis of **DTNIF**: To a solution of Compound **1** (174 mg, 0.2 mmol) in dry CHCl_3_ (30 mL), 2-(6-fluoro-3-oxo-2,3-dihydro-1H-inden-1-ylidene)malononitrile (337 mg, 1.6 mmol) were added. After degassing with nitrogen for 30 min, 1 mL of pyridine was added into the mixture which was further stirred at reflux for 24 h under nitrogen atmosphere. Then the mixture was cooled down to room temperature. The reaction mixture was poured into 100 mL of methanol. A precipitate was formed and filtered off which was further purified by using column chromatography (silica gel) with petroleum ether/CH_2_Cl_2_ (1:1) as the eluent. A dark metallic luster solid (126 mg, 60%) was obtained. ^1^H NMR (CDCl_3_, 400 MHz, ppm): 9.07 (d, *J* = 8.0 Hz, 2H), 8.79–8.76 (m, 0.8H), 8.46–8.43 (m, 3.2H), 8.04–8.00 (m, 1.3H), 7.93–7.88 (m, 2H), 7.85–7.80 (m, 2H), 7.67–7.64 (m, 0.7H), 7.51–7.46 (m, 2H), 2.23–2.11 (m, 8H), 1.00–0.52 (m, 60H). HRMS (MALDI) m/z: calc. for C_78_H_82_F_2_N_4_O_2_S_2_: 1,209.5891; found: 1,209.5920. Elemental analysis (%) calc. for C_78_H_82_F_2_N_4_O_2_S_2_: C, 77.45; H, 6.83; N, 4.63; found: C, 77.50; H, 7.26; N, 4.24.

### OSC fabrication and characterization

PSCs were fabricated by using a device configuration of indium tin oxide (ITO)/ZnO/active layer/MoO_3_/Ag. The ITO glass was cleaned by sequentially in detergent, deionized water, acetone, and isopropanol for half an hour each and dried for more than 12 h in an oven. Then, the ITO glass was subjected to ultraviolet/ozone treatment for 15 min. Later on, a ZnO precursor solution (0.23 M in 2-methoxyethanol) was spin-coated on the ITO glass at 3,000 rpm for 50 s. On a hot plate (130°C), the obtained films were heated for 10 min first, then they were annealed by an oven (200°C) for 1 h. The blend ratios of the active layer (PBDB-T:acceptor) were fixed at 1:1 by weight. The donor/acceptor blends were dissolved in chlorobenzene with a total concentration of 18 mg/mL and spin-coated on top of the ZnO film (ca. 30 nm) in the glovebox. Finally, on the active layer, 10 nm of MoO_3_ film was deposited followed by a further deposition of Ag film (100 nm). The active area of OSC devices was 6 mm^2^.

Solar cell characterization was tested under AM 1.5 G irradiation (100 mW cm^−2^) from an Oriel Sol3A simulator (Newport) with a NREL-certified silicon reference cell. *J*–*V* measurements were carried out in air using a Keithley 2440 source measurement unit. External quantum efficiency (EQE) data were collected by a Newport EQE measuring system.

### Instruments and measurements

^1^H NMR was measured in CDCl_3_ on a Bruker AVANCE-spectrometer. Elemental analysis of the non-fullerene acceptors was obtained on an Elementar Vario EL Cube analyzer. UV–Vis absorption spectra for all the samples were performed on a Perkin-Elmer Lambda 365 spectrophotometer. Linear emission spectra for pure films or blended films were obtained by using a FLS920 spectrophotometer. Atomic force microscopy (AFM) was conducted in a tapping mode with a Bruker Nanoscale V station. A Bruker Dektak XT surface profilometer was used to test the thickness of thin films in this work. A three-electrode CHI 604E electrochemical workstation was used to run the cyclic voltammetry (CV) using Bu_4_NPF_6_ solution (0.1 M in acetonitrile) and a scan rate of 100 mV s^−1^. The solid films were precipitated on a Pt plate through dipping the Pt plate into the corresponding chloroform solutions and then took out for drying. Ag/AgNO_3_ and a Pt wire were chosen as the reference electrode and the counter electrode, respectively. The LUMO and HOMO energy levels of films made by the small molecule were calculated by using the following equations:

$$\begin{array}{l} E_{\text{HOMO}}=-\left(\varphi_{\text{ox}}+4.82\right)\left(\text{eV}\right)\\ E_{\text{LUMO}}=-\left(\varphi_{\text{RED}}+4.82\right)\left(\text{eV}\right) \end{array}$$

Agilent 4155C semiconductor parameter analyzer was used to conduct the mobility measurements. Electron and hole mobilities were determined by using the space charge limited current model (SCLC) with an electron-only diode configuration of ITO/ZnO/active layer/Ca/Al and an hole-only diode configuration of ITO/PEDOT:PSS/active layer/Au, using current-voltage measurements in the range of –(3–10) V in the dark. The SCLC mobility was calculated by fitting the *J-V* curves to the Mott-Gurney relationship:

$$\begin{array}{l} J=\frac{9}{8}\varepsilon_{r}\varepsilon_{0}\mu \frac{V^{2}}{L^{3}} \end{array}$$

Where ε_0_ is the permittivity of free space (8.85 × 10^−12^ F m^−1^), ε_r_ is the dielectric constant of the active layer material (assumed to be 3), μ is the electron or hole mobility, *L* is the active layer thickness, *V* is the voltage drop across the electron- or hole-only device (*V*_appl_ – *V*_bi_, where *V*_appl_ is the applied voltage, and *V*_bi_ is the built-in voltage induced by the work function difference of the two electrodes). The electron/hole mobilities can be determined according to the slope of the *J*^1/2^–*V* curves.

## Author contributions

QZ conceived the experiments. MZ and YM were primarily responsible for the experiments. MZ and QZ wrote the manuscript. All authors discussed the results.

### Conflict of interest statement

The authors declare that the research was conducted in the absence of any commercial or financial relationships that could be construed as a potential conflict of interest.
